# Immunisation efficacy of a stabilised SARS-CoV-2 spike glycoprotein in two geriatric animal models

**DOI:** 10.1038/s41541-024-00840-0

**Published:** 2024-02-27

**Authors:** Carla Usai, Erola Ainsua-Enrich, Victor Urrea Gales, Edwards Pradenas, Cristina Lorca-Oró, Ferran Tarrés-Freixas, Núria Roca, Mónica Pérez, Carlos Ávila-Nieto, María Luisa Rodríguez de la Concepción, Núria Pedreño-Lopez, Julieta Carabelli, Benjamin Trinité, Ester Ballana, Eva Riveira-Muñoz, Nuria Izquierdo-Useros, Bonaventura Clotet, Julià Blanco, Victor Guallar, Guillermo Cantero, Júlia Vergara-Alert, Jorge Carrillo, Joaquim Segalés

**Affiliations:** 1 Unitat Mixta d’Investigació IRTA-UAB en Sanitat Animal, Centre de Recerca en Sanitat Animal (CReSA), Campus de la Universitat Autònoma de Barcelona (UAB), Bellaterra, Spain; 2 IRTA, Programa de Sanitat Animal, CReSA, Campus de la Universitat Autònoma de Barcelona (UAB), Bellaterra, Spain; 3 IrsiCaixa AIDS Research Institute, Badalona, Spain; 4 Germans Trias i Pujol Research Institute (IGTP), Campus Can Ruit, Badalona, Spain; 5 CIBERINFEC. ISCIII, Madrid, Spain; 6 Centre for Health and Social Care Research (CESS), Faculty of Medicine, University of Vic – Central University of Catalonia (UVic – UCC), Vic, Catalonia Spain; 7 Fundació Lluita contra les Infeccions, Badalona, Spain; 8 Life Science Department, Barcelona Supercomputing Center (BSC), Barcelona, Spain; 9 Catalan Institution for Research and Advanced Studies, Barcelona, Spain; 10 Department de Sanitat i Anatomia Animals, Facultat de Veterinària, Campus de la UAB, Bellaterra, Spain

**Keywords:** Respiratory tract diseases, Experimental models of disease

## Abstract

Age is associated with reduced efficacy of vaccines and linked to higher risk of severe COVID-19. Here we determined the impact of ageing on the efficacy of a SARS-CoV-2 vaccine based on a stabilised Spike glycoprotein (S-29) that had previously shown high efficacy in young animals. Thirteen to 18-month-old golden Syrian hamsters (GSH) and 22–23-month-old K18-hCAE2 mice were immunised twice with S-29 protein in AddaVax^TM^ adjuvant. GSH were intranasally inoculated with SARS-CoV-2 either two weeks or four months after the booster dose, while all K18-hACE2 mice were intranasally inoculated two weeks after the second immunisation. Body weight and clinical signs were recorded daily post-inoculation. Lesions and viral load were investigated in different target tissues. Immunisation induced seroconversion and production of neutralising antibodies; however, animals were only partially protected from weight loss. We observed a significant reduction in the amount of viral RNA and a faster viral protein clearance in the tissues of immunized animals. Infectious particles showed a faster decay in vaccinated animals while tissue lesion development was not altered. In GSH, the shortest interval between immunisation and inoculation reduced RNA levels in the lungs, while the longest interval was equally effective in reducing RNA in nasal turbinates; viral nucleoprotein amount decreased in both tissues. In mice, immunisation was able to improve the survival of infected animals. Despite the high protection shown in young animals, S-29 efficacy was reduced in the geriatric population. Our research highlights the importance of testing vaccine efficacy in older animals as part of preclinical vaccine evaluation.

## Introduction

Severe acute respiratory syndrome coronavirus 2 (SARS-CoV-2) is a highly pathogenic respiratory virus that emerged in China at the end of 2019^1^. It rapidly spread worldwide causing the Coronavirus disease 2019 (COVID-19), which was declared a Public Health Emergency of International Concern in January 2020 by the World Health Organization (WHO), and later characterised as a pandemic in March 2020^2,3^. The tremendous impact of COVID-19 on public health led to an unprecedented global effort for the development of diagnostic, preventive, and therapeutic interventions^4,5^.

As the elderly population was severely hit by the first wave of the COVID-19 pandemic, age was immediately identified as one of the main risk factors for the development of severe COVID-19^6,7^. Data collected by the Centers for Disease Control and Prevention (CDC) show that the hospitalisation rate for COVID-19 in American people between 65 to 74 years of age was five times higher than the 18–29-year-old age group (chosen as the reference group since it accounts for the largest cumulative number of cases). The death rate of the elderly population was 60 times higher. These rates further increased with age, reaching a 350 times greater rate of death for COVID-19 patients older than 85 years^8^. For this reason, the vaccine rollout that started in December 2020 in several countries worldwide, prioritised the immunisation of the elderly as well as people with comorbidities^9^. However, new SARS-CoV-2 variants appeared later on showing higher transmissibility, pathogenicity, and resistance to neutralising antibodies (nAbs) elicited by previous natural infection or vaccination. This, and the waning of antibody titres over time, particularly in the elderly population, prompted to improve vaccines immunogenicity and efficacy by adapting either their antigens to new variants or the immunisation protocols. As of June 2023, up to 4 doses have been offered to the over-65-year-old population in many countries^10^.

It is known that ageing is associated with immunosenescence, a phenomenon characterised by an impaired ability to respond to new antigens, unsustained memory responses, greater propensity for autoimmunity, and persistent low-grade inflammation^11,12^. Moreover, differences in the efficacy of vaccination for several infectious diseases between ages have been described^13–17^, including COVID-19^18–21^. Ageing is therefore a determining factor in the evaluation of vaccine efficacy in the overall population. To date, however, a limited number of preclinical studies of COVID-19 vaccines using geriatric animal models have been published, and, to our knowledge, only two of them offer post-immunisation data from animals that were subsequently challenged with SARS-CoV-2^22–25^.

Here we tested the immunogenicity and efficacy of a stabilised Spike glycoprotein (S-29) (K986P/V987P/S758E/T912R/K947R), with improved production (five-fold) when compared with the S2-P (K986P/V987P)^26^, in protecting from SARS-CoV-2-induced disease after viral challenge in two geriatric animal models: golden Syrian hamsters (GSH) and K18-human ACE2 transgenic (K18-hACE2-tg) mice. To this end, we chose a population of extremely old mice (660–690 days old; C57BL/6 median lifespan 866 days (females) and 901 days (males)^27^), close to the maximum limit of their lifespan^28^ as well as hamsters between one and two years of age (average lifespan two years^29^). In addition, we tested the duration of immunisation-induced immunity in GSH, performing the viral challenge at two different time points after the booster dose. Despite the S-29 core sequence being based on the ancestral WH1 strain, this immunogen had proved effective in young adult animals, protecting them from severe disease upon experimental inoculation with the highly pathogenic and neutralisation-resistant SARS-CoV-2 Beta variant^26^.

## Results

### S-29 is immunogenic and improves the survival of geriatric K18-hACE2 mice upon SARS-CoV-2 inoculation

**Fig. 1:**
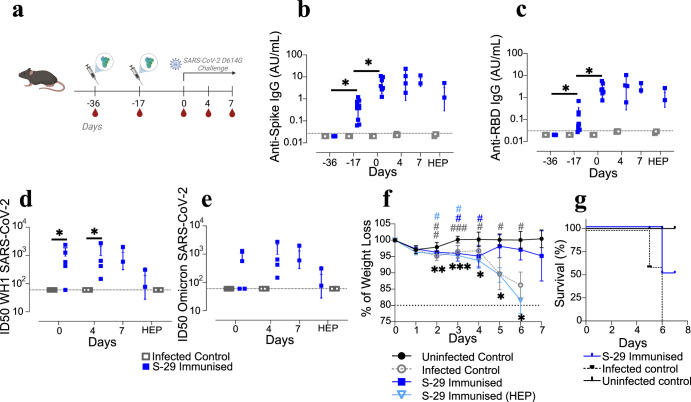
Study design, survivability, weight evolution and humoral immune responses of SARS-CoV-2 inoculated K18-hACE2 geriatric mice. **a** Overview of the immunisation and inoculation strategy in K18-hACE2 geriatric mice. Animals received two immunisation doses, three weeks apart (syringe symbol); blood drops indicate the collection of biological samples, SARS-CoV-2 D614G inoculation/challenge two weeks after the booster dose. Kinetics of the humoral immune response in K18-hACE2 geriatric mice: anti-S IgG (**b**), anti-RBD IgG (**c**); neutralising activity against SARS-CoV-2 WH1 (**d**) and Omicron BA.1 (**e**) variants after viral challenge (pseudovirus neutralisation assay). Differences between groups were determined using the Kruskal-Wallis test. *P* values < 0.05 were considered significant: *. **f** Body weight was recorded daily after inoculation. Differences between groups were assessed by mixed two-way ANOVA followed by Tukey’s multiple comparisons test, using the infected control or the uninfected control group as a reference; statistically significant differences are indicated by the * or # symbol, respectively, depicted with the colour of the group being different. Mean and standard deviation are shown for each experimental group. *P* values < 0.05 were considered significant; *, #: *p* value < 0.05; **, ##: *p* value < 0.005; ***, ###: *p* value < 0.001. **g** Kaplan-Meier plot showing the survival rate after inoculation.

The immunogenicity and efficacy of the S-29 immunogen were evaluated in geriatric K18-hACE2 mice. Animals were immunised twice (three weeks apart) and inoculated with the SARS-CoV-2 D614G variant two weeks after booster immunisation. Mice were monitored until 7 days post-inoculation (dpi); body weight and clinical signs were recorded daily (Fig. 1a).

Anti-S and anti-RBD IgG were detected after the first immunisation, and their levels increased significantly after receiving the booster dose, reaching a plateau post-viral inoculation (Fig. 1b, c). However, their antibody levels were lower than those elicited in young animals (Supplementary Figure 1a, b). Neutralising activity against both SARS-CoV-2 WH1 and Omicron BA.1 variants from immunised and non-immunised inoculated mice were assessed at 0, 4 and 7 dpi using a pseudovirus neutralisation assay. Non-immunised mice did not show neutralising activity at any time point against the SARS-CoV-2 tested variants. However, S-29-immunised mice developed nAbs against the WH1 variant from 0 dpi, and until the end of the study. None of the non-immunised and only one out of two immunised animals that were euthanised before the allocated time point had developed detectable nAbs against Omicron BA.1 (Fig. 1d, e).

Animals from the infected control group showed a progressive body weight loss, compatible with SARS-CoV-2 infection and disease progression, that was significantly different from the uninfected control group at all time points. Weight variation in 2 out of 4 immunised animals was similar to that of the infected controls during the entire study period, and significantly different from the uninfected control mice at 3 and 4 dpi. Interestingly, the other half of the immunised animals started recovering at 5 dpi, and survived until the end of the study (Fig. 1f, g). On the contrary, all the infected controls succumbed to the infection and were euthanized by 5-6 dpi (Fig. 1g).

### Immunisation of K18-hACE2 mice with S-29 reduces the amount of sgRNA in respiratory tissues

**Fig. 2:**
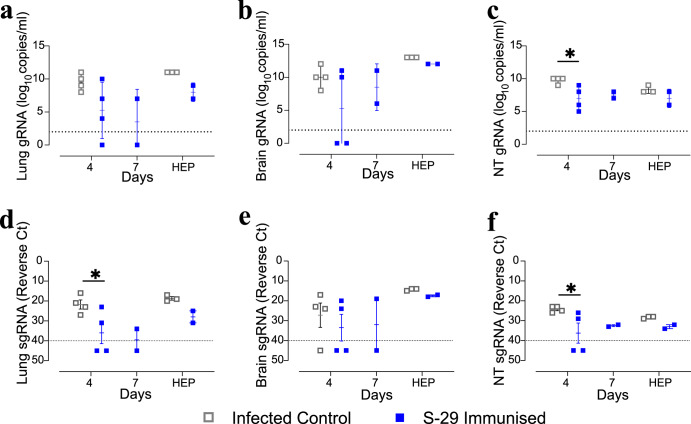
Viral load in K18-hACE2 geriatric mice tissues. Quantification of gRNA and sgRNA in lung (**a**, **d**), brain (**b**, **e**), and nasal turbinates (**c**, **f**) samples from SARS-CoV-2 inoculated K18-hACE2 geriatric mice. Levels of SARS-CoV-2 gRNA are expressed as log_10_ of copies per mL of tissue sample lysate; levels of sgRNA are expressed as the Ct value of each sample. The dotted lines represent the limit of quantification (LOQ) of the specific RT-qPCR protocol (10^2^ copies/ml or Ct = 40, respectively). Differences between groups were analysed using the Peto&Peto left-censored samples test; *P* values < 0.05 were considered significant: * NT nasal turbinate.

The presence and replication of SARS-CoV-2 were determined by RT-qPCR targeting the genomic (gRNA) and subgenomic (sgRNA) RNA, respectively, in the lungs, nasal turbinates (NT), and brain. No differences were detected in the levels of gRNA in the lungs and brain between immunised and non-immunised mice, as they remained stable over time (Fig. 2a, b). However, lower amounts of gRNA in the NT of immunised mice 4 dpi were detected (Fig. 2c). Unlike gRNA, the levels of sgRNA decreased in all sample types at 4dpi, except for the brain (Fig. 2d–f).

### S-29 immunisation reduce infectious SARS-CoV-2 shedding in NT and lung from K18-hACE2 mice

**Fig. 3:**
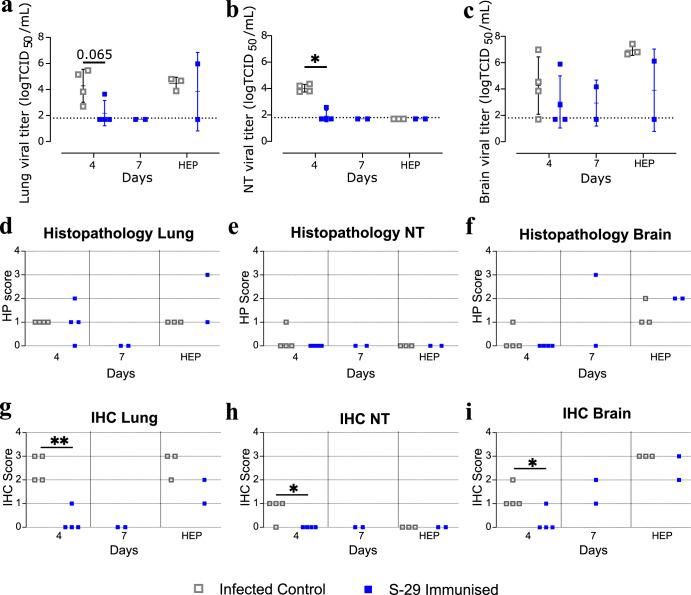
SARS-CoV-2 titration, histopathology and immunohistochemistry to detect viral antigen of K18-hACE2 geriatric mice tissues. **a**–**c** Determination of infectious viral particles (IVP) in lung, nasal turbinate (NT) and brain samples from SARS-CoV-2 inoculated K18-hACE2 geriatric mice after immunisation. Supernatants from the homogenized lungs (**a**), nasal turbinates (**b**) and brain (**c**) were evaluated for the presence of infectious virus by titration in Vero E6 cells. Viral titres are expressed as the TCID_50_ per mL of tissue lysate. The dotted lines represent the limit of quantification (LOQ) of the Reed-Muench method. Single data points, mean and standard deviation are shown for each group. Differences between groups were analysed using the Peto&Peto left-censored samples test. *P* values < 0.05 were considered significant: *. **d**–**i** Histopathological assessment and detection of SARS-CoV-2 nucleoprotein (NP) by IHC in tissue samples from SARS-CoV-2 inoculated K18-hACE2 geriatric mice. The lesion severity and extent in lung (**d**), nasal turbinate (**e**), and brain (**f**) samples were expressed using a semi-quantitative score: 0 = none, 1 = mild, 2 = moderate, 3 = severe. The presence and amount of viral antigen in lung (**g**), nasal turbinates (**h**), and brain (**i**) samples were assessed by immunohistochemistry (IHC) using a semi-quantitative score: 0 = absence of viral antigen; 1 = low amount, multifocal localisation; 2 = moderate amount, multifocal localisation; 3 = high amount, diffuse localization. Differences between groups were analysed using an asymptotic generalized Pearson chi-squared test; multiple comparisons were corrected by false discovery rate (FDR). *P* values < 0.05 were considered significant; *p value < 0.05; **p value < 0.005. NT : nasal turbinate.

After analyzing the presence of gRNA and sgRNA, we determined the presence of infectious viral particles (IVP) in the same samples. IVP loads in lung tissue were reduced in the immunised group by 4 dpi. All positive control mice and one vaccinated mouse that reached HEP showed elevated levels of IVP (Fig. 3a). In NT, there was a significant reduction of IVP in the S-29 immunised group compared to the control group at 4 dpi. All animals from both groups had undetectable IVP after this time point (Fig. 3b). No IVP load differences were observed between immunised and non-immunised groups in brain tissue (Fig. 3c).

### Immunisation reduces the severity of lung lesions in surviving K18-hACE2 mice and improves viral clearance at early time points

Both immunised and non-immunised mice developed from none to mild or moderate multifocal broncho-interstitial pneumonia by 4 dpi. By 7 dpi only two immunised mice survived, and no evidence of lung lesions was observed. The infected control mice reaching humane endpoint (HEP) displayed mild lesions, while the two immunised animals that reached HEP displayed mild or severe pneumonia (Fig. 3d). No significant NT lesions were observed in any of the animals, except for a mild non-suppurative multifocal rhinitis in one positive control at 4 dpi (Fig. 3e). Brain lesions consisting of non-suppurative meningo-encephalitis were observed in one positive control mouse at 4 dpi, in one immunised mouse at 7 dpi, and in all animals reaching HEP (Fig. 3f).

As a proxy of viral replication, we determined the levels of nucleoprotein (NP) by immunohistochemistry (IHC). Higher levels of SARS-CoV-2 NP were found in lungs, NT, and brain of infected control mice at 4 dpi (Fig. 3g–i). Low levels of pulmonary immunolabelling were detected in just one immunised animal at 4 dpi and mild to moderate in the ones reaching HEP. No viral antigen was detected in the lungs of immunised mice at 7 dpi (Fig. 3g). SARS-CoV-2 NP was detected in three out of four non-immunised animals by 4 dpi in NT. In contrast, NP was not detected in NT of immunised mice by this time point. No NP was detected in NT samples from both immunised and non-immunised groups at later time points (Fig. 3h). Finally, mild to moderate viral immunolabelling was observed in brain samples from infected control mice by 4 dpi, while this group showed high levels of viral antigen at HEP. In contrast, only one immunised mouse had limited SARS-CoV-2 NP staining by 4 dpi, while it was moderate to high in those reaching HEP and mild to moderate in the ones euthanized at 7 dpi (Fig. 3i).

### S-29 is immunogenic and protects geriatric GSH from progressive weight loss upon SARS-CoV-2 inoculation

**Fig. 4:**
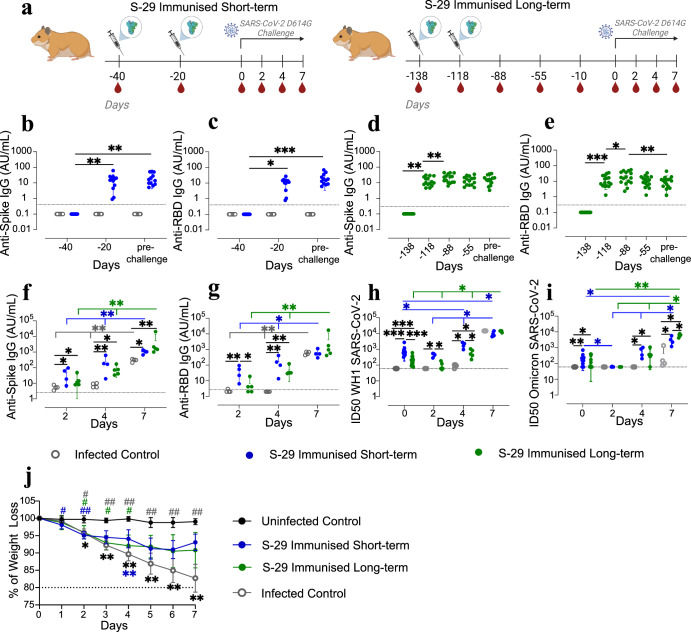
Study design, weight evolution and humoral immune responses of SARS-CoV-2 inoculated golden Syrian hamsters (GSH). **a** Overview of the immunisation and inoculation strategies in geriatric GSH. Animals received two immunisation doses, three weeks apart (syringe symbol); blood drops indicate the collection of biological samples; GSH were inoculated either 20 days or 4 months after the booster dose. **b**–**i** Kinetics of the humoral immune response in geriatric GSH: anti-S IgG and anti-RBD IgG in both the short-term and long-term groups before (**b**–**e**) and after (**f**, **g**) inoculation; neutralising activity against SARS-CoV-2 WH1 (**h**) and Omicron BA.1 (**i**) variant after inoculation (pseudovirus neutralisation assay). Differences between groups were determined using the Kruskal-Wallis test, and longitudinally using the Friedman test. Post-hoc tests were performed using the corresponding Conover’s test. *P* values < 0.05 were considered significant; *p value < 0.05; **p value < 0.01; ***p value < 0.001. **j** Body weight was recorded daily after inoculation. Differences between groups were assessed by mixed two-way ANOVA followed by Tukey’s multiple comparisons test, using the infected control or the uninfected control group as a reference; statistically significant differences are indicated by the * or # symbol, respectively, depicted with the colour of the group being different. Mean and standard deviation are shown for each experimental group. *P* values < 0.05 were considered significant; *, # *p* value < 0.05; **, ## *p* value < 0.005.

To confirm K18-hACE2 mice results, we performed an immunisation and challenge study in geriatric GSH. GSH received two S-29 doses with a three-week interval. In addition, to investigate whether time from booster dose impacts the efficacy of the vaccine, immunised animals were inoculated with SARS-CoV-2 at two different time points (Fig. 4a). Twelve GSH were challenged with SARS-CoV-2 D614G 20 days after the booster dose when the levels of anti-S and anti-RBD were still rising (short-term group) (Fig. 4b, c); 15 GSH were challenged 4 months after the booster (long-term group) when the levels of anti-RBD IgG had already started fading while the anti-S IgG levels were stable (Fig. 4d, e). Immunised geriatric GSH had detectable levels of anti-S and anti-RBD antibodies that slightly increased after receiving the booster dose (Fig. 4b–e). Interestingly, their kinetics was delayed compared with young animals (Supplementary Figure 1c, d). Geriatric GSH in both immunised groups experienced an increase in anti-S IgG levels after the challenge, which continued until the end of the study, being significantly higher than the levels in the infected controls at any time point (Fig. 4f). Similarly, the levels of anti-RBD IgG increased in both immunised groups after the challenge, being significantly higher than the levels of the infected control group, which were undetectable at 2 and 4 dpi. At 7 dpi, however, anti-RBD IgG levels of non-immunised animals reached the same values as the immunised groups (Fig. 4g). In both the short- and long-term groups, the levels of anti-S and anti-RBD IgG increased during the entire study period (Fig. 4f, g). SARS-CoV-2 WH1 and Omicron BA.1 neutralising activity of sera was assessed from either immunised and non-immunised inoculated GSH at 0, 2, 4, and 7 dpi using a pseudovirus neutralisation assay. Non-immunised animals did not show neutralising activity against the tested SARS-CoV-2 variants until 7 dpi; immunised GSH developed nAbs both against the homologous and Omicron BA.1 variants. In the short-term group, homologous nAbs were detected two days after the challenge, and their levels were higher than both non-immunised control and long-term groups (Fig. 4h). The neutralising activity of the long-term group started to be consistently detectable at 4 dpi, being significantly lower than the short-term group. By the end of the experiment, both the immunised and the non-immunised groups had comparable neutralisation titres against the WH1 variant (Fig. 4h). The production of nAbs against Omicron appeared later in all experimental groups. However, these antibodies are detected earlier in immunised animals (4 dpi) than in controls (7 dpi) (Fig. 4i). In contrast with the homologous neutralising activity, by the end of the study period, the titres of nAbs against Omicron were higher in the long-term than in the short-term group, the latter being not significantly different from the titres of non-immunised animals (Fig. 4i). In both the short- and long-term groups, neutralising titres increased over time (Fig. 4h, i).

Body weight and clinical signs were recorded daily after the viral challenge and until 7 dpi (Fig. 4a, j). Average weight of all inoculated groups started to significantly decrease as soon as 1 dpi. The difference with the non-inoculated group became statistically non-significant by 3 and 5 dpi for the short- and long-term groups, respectively. Animals from the infected control group had the lowest average weight, which was significantly lower than the uninfected controls from 2 dpi onward. No difference was recorded between the positive controls and long-term immunised animals due to the high intra-group variability. Similarly, a statistically significant difference between the infected controls and the short-term group was observed only at 4 dpi. Weight recovery was observed in both immunised groups at 6-7 dpi (Fig. 4j). None of the animals lost 20% or more of the initial weight or reached the pre-established HEP.

### Immunisation-to-inoculation intervals impact gRNA amount in nasal turbinates and lungs of GSH

**Fig. 5:**
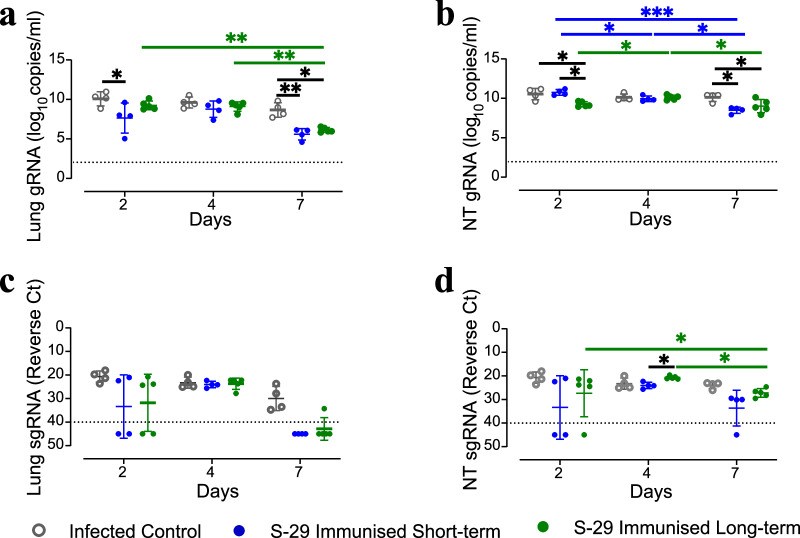
Viral load in geriatric golden Syrian hamster tissues. Quantification of gRNA and sgRNA in lungs (**a**, **c**) and nasal turbinates (**b**, **d**) samples from SARS-CoV-2 inoculated geriatric GSH. Levels of SARS-CoV-2 gRNA are expressed as log_10_ of copies per mL of tissue sample lysate; levels of sgRNA are expressed as the Ct value of each sample. The dotted lines represent the limit of detection quantification (LOQ) of the specific RT-qPCR protocol (10^2^ copies/ml or Ct = 40, respectively). Differences between groups were analysed using the Peto&Peto left-censored samples test*. P* values < 0.05 were considered significant; * *p* value < 0.05; ** *p* value < 0.01; *** *p* value < 0.001. NT nasal turbinate.

The presence and replication of SARS-CoV-2 were determined by RT-qPCR targeting the gRNA and sgRNA in respiratory tissue samples. Inoculation after a short period post-immunisation corresponded to a lower amount of gRNA in the lungs compared to non-immunised hamsters at 2 dpi. Lower gRNA levels were detected in the NT of the long-term group, while both immunised groups showed decreased gRNA levels than the infected controls in lungs and NT at 7 dpi (Fig. 5a, b).

When sgRNA was quantified, no statistically significant differences were found among groups at any time point, possibly because of the intra-group variability (Fig. 5c, d).

### S-29-immunisation does not alter SARS-CoV-2 ability to cause productive infection upon inoculation of geriatric GSH

**Fig. 6:**
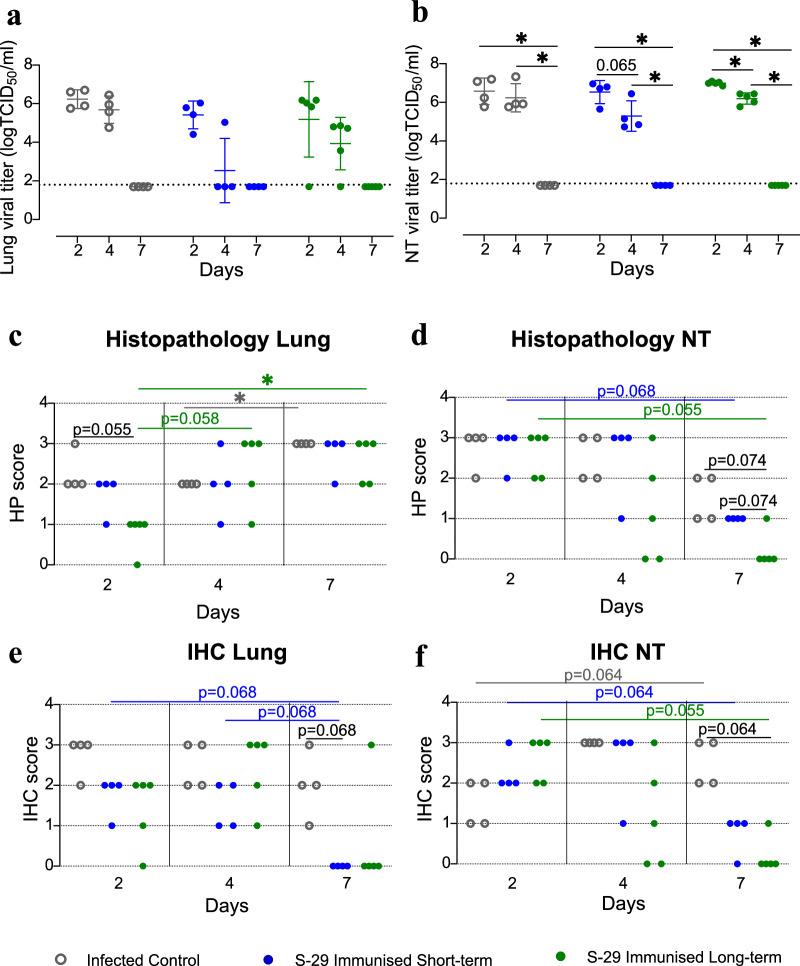
SARS-CoV-2 titration, histopathology and immunohistochemistry to detect viral antigen of geriatric golden Syrian hamster (GSH) tissues. **a**, **b** Infectious viral particle quantification in lung and nasal turbinate samples from SARS-CoV-2 inoculated GSH after vaccination. Supernatant from the homogenized lungs as nasal turbinates were evaluated for the presence of infectious virus by titration in Vero E6 cells. Viral titres are expressed as the TCID_50_ per mL of tissue lysate. The dotted lines represent the limit of quantification (LOQ) of the Reed-Muench method. Single data points, mean and standard deviation are shown for each group. Differences between groups were analysed using the Peto&Peto left-censored samples test. *P* values < 0.05 were considered significant: * NT: nasal turbinate. **c**–**f** Histopathological assessment and detection of SARS-CoV-2 nucleoprotein by IHC in tissue samples from SARS-CoV-2 inoculated geriatric hamsters. The degree of inflammation as well as lesion severity and extent in lungs (**c**) and nasal turbinates (**d**) were expressed using a semi-quantitative score: 0 = none, 1 = mild, 2 = moderate, 3 = severe. The presence and amount of SARS-CoV-2 nucleoprotein (NP) in lung (**e**) and nasal turbinate (**f**) samples were assessed by IHC; each sample was assigned a semi-quantitative score: 0 = absence of viral antigen; 1 = low amount, multifocal localisation; 2 = moderate amount, multifocal localisation; 3 = high amount, diffuse localization. Differences between groups were analysed using an asymptotic generalized Pearson chi-squared test; multiple comparisons were corrected by false discovery rate (FDR). *P* values < 0.05 were considered significant: * NT nasal turbinate.

Besides the detection of sgRNA and NP by IHC (see below), the occurrence of active infection was determined by the isolation of infectious viral particles (IVP) from respiratory tissues. A faster-decreasing trend in the levels of IVP was observed in NT and lung samples from immunised animals. Particularly, the IVP reduction was more evident for the short-term group at 4 dpi in lung tissue (Fig. 6a, b). On the other hand, a significant decrease over time was observed within all groups in NT samples (Fig. 6b). Infectious viral particles were undetectable or below the limit of quantification (LOQ) at 7 dpi in all samples from inoculated animals, irrespectively of their immunisation status (Fig. 6a, b).

### Immunisation enhances viral protein clearance in NT and lungs of GSH

For all groups, lesions lasted longer in lung samples (multifocal broncho-interstitial pneumonia) than in NT (muco-purulent rhinitis), and their severity increased over time. Most lung samples were assigned the highest score by the end of the study period (Fig. 6c, d). On the contrary, a decrease in the lesion severity was observed in NT over time in all groups. Half of the samples from non-immunised animals had a score of 2, and the other half, a score of 1 at 7 dpi. Immunisation had a stronger effect on the NT lesion score when inoculation was performed after a longer interval: 2 out of 5, and 4 out of 5 animals had no visible lesions at 4 and 7 dpi, respectively, while all GSH in the short-term group still had visible lesions at the end of the study period (Fig. 6d). Immunisation did not exert any effect on the lesions in the lungs, which were assigned the highest score in the majority of short- and long-term samples, and in all non-immunised hamsters (Fig. 6c).

In terms of NP detection, the effect of the immunisation was more noticeable in the lungs, where only one animal from the long-term group and none from the short-term group stained positive for SARS-CoV-2 NP at 7 dpi (Fig. 6e). Both immunisation-to-inoculation intervals reduced the number of animals with detectable SARS-CoV-2 NP in the analysed tissues. Moreover, in those animals with detectable levels, scores were overall lower in the vaccinated groups compared to the infected controls. In the NT, the effect seemed stronger after the longer interval, since only 1 out of 5 hamsters had detectable viral antigen and with the lowest score, while in the short-term group, the labelling was still present in most animals (Fig. 6f).

## Discussion

Life extension of the world population is leading to an increase in age-associated diseases, including a higher susceptibility to infections. This issue became evident during the early phase of the COVID-19 pandemic, when the elderly population was severely hit^6,30^. As soon as the first SARS-CoV-2 vaccines became available, the elderly, immunosuppressed and people with comorbidities were prioritised^9^. Importantly, different responsiveness to vaccination across age groups had been reported for several infectious diseases, with COVID-19 being no exception^18–20^. Immunosenescence is associated with ageing and is characterised by the diminished ability of the immune system to react to new antigens and vaccines. Even though the molecular mechanisms behind immunosenescence are not yet fully characterised, increasing evidence shows that dysfunctions of both the innate and the adaptive arms of the immune system are involved in this process^31^.

In the present work, we investigated the immunogenicity (humoral responses) and efficacy of a stabilised Spike glycoprotein (S-29) adjuvanted with AddaVax^TM^ in geriatric individuals of two well-established animal models of SARS-CoV-2 infection: K18-hACE2 mice and GSH. For the latter, we also tested the durability of the vaccination-induced protection by inoculating them either three weeks (short-term) or four months (long-term) after the booster dose. Our experimental immunogen consists of a trimeric S-2P glycoprotein (K986P/V987P)^32^ further stabilised in its pre-fusion conformation by non-proline mutations (S758E/T912R/K947R). S-29 has already proved effective in young adults of both species, protecting them from severe disease^26^. In addition, since MF59 adjuvant is included in vaccines for the elderly^13^, we used an MF59-like adjuvant (AddaVax^TM^) in our formulation.

Like humans, older animals develop a more severe form of the disease than young individuals upon SARS-CoV-2 infection^33–35^. S-29 immunisation improved the survival rate of geriatric mice upon SARS-CoV-2 inoculation, and partially protected both species from progressive weight loss, indicating a less severe clinical-pathological outcome. The immunogenicity of the S-29-based product was indicated by the production of anti-S and anti-RBD IgG and confirmed in all immunised animals after the booster dose, which reached a plateau upon inoculation in mice. IgG levels continued to increase in the post-challenge period in GSH. Interestingly, whereas only one immunised K18-hACE2 mouse displayed neutralising activity against Omicron, all immunised GSH were able to neutralise the heterologous variant in vitro within a week after the experimental inoculation. This result suggests a difference in reactivity between the two species, rather than an intrinsic characteristic of the immunogen. In fact, non-immunised hamsters, but not mice, seroconverted and produced nAbs upon inoculation (although at a slower pace than immunised ones).

In addition, the kinetics of the development of anti-S and anti-RBD IgG was similar between young adult and geriatric K18-hACE2 mice, with the latter eliciting significantly lower levels. On the other hand, anti-S and anti-RBD IgG produced by geriatric GSH followed the same rate, but at lower levels than younger animals. However, after the challenge, GSH developed a faster humoral response against the virus, not observed in K18-hACE2 mice, which may explain why GSH developed a moderate disease upon SARS-CoV-2 challenge, whereas K18-hACE2 mice succumbed to the disease.

From the virological point of view, the detection and infectivity of SARS-CoV-2 D614G were not prevented by immunisation, which is expected since our approach was not designed to induce sterilising immunity but systemic immune responses. Nevertheless, a significant reduction in the amount of viral gRNA was observed in the NT of immunised GSH and the lungs of those inoculated shortly after the booster (short-term group). Similarly, immunisation reduced detectable gRNA in the NT of mice, and sgRNA in all tissues, except for the brain (consistent with the severe neuroinvasion previously described in this model^36^). Importantly, viral RNA clearance was not achieved by 7 dpi despite immunisation, and viral replication was not halted in this model. However, the kinetics of infectious viral particle clearance was faster in the respiratory tissues of vaccinated GSH. Furthermore, NP-specific staining was significantly reduced in the lungs, NT, and brain of the surviving immunised mice 4 dpi. A slight reduction was observed in hamsters. Consistently, tissue lesions were mild to moderate in the lungs of surviving immunised mice at the same time point, with no visible lesions in the NT. A mild but not significant protective effect was observed in the respiratory organs of immunised GSH. In the case of geriatric GSH, a shorter interval between the booster dose and the inoculation induced a humoral response with higher nAbs levels against the homologous variant, and viral load reduction was particularly evident in the lungs.

Other studies have shown that GSH phenocopy human age-dependent differences in both the course of SARS-CoV-2 infection and the associated immune response, while viral replication appeared not to be age-related, which is consistent with our results^35,37^. Both papers, based on much younger animals than those used in our work, showed similar levels of antibody production between younger and older hamsters after SARS-CoV-2 infection; nevertheless, the neutralising activity was significantly reduced in the older cohorts. The poorer humoral response shown by both aged K18-hACE2 mice and GSH when compared with young animals^26^ probably contributed to the lack of full protection in this animal model. Moreover, those mice that reached the humane end point showed the lowest titres of SARS-CoV-2 neutralizing antibodies on the day of euthanasia. However, in contrast with Osterreider and collegues^35^, we were able to detect neutralising activity in the infected group at earlier time point post-inoculation.

Unlike other research groups, we were not able to investigate other aspects of the innate and adaptive immune responses (such as expansion and functionality of virus-specific T and B cell populations) due to logistic limitations of the BSL3 facilities where the experiments were performed. Further analyses in these directions would be useful to characterise the mechanisms behind the observed age-related differences as well as to potentially develop ad hoc products and vaccination strategies for elderly subjects.

Despite the number of animals included in each experimental group was low due to difficulties of obtaining advanced-age animals (particularly in the mice group), we can conclude that a prime-boost administration of adjuvanted S-29 in our advanced geriatric models ameliorated some of the clinical signs in K18-hACE2-mice and GSH, and improved survival in mice, but did not prevent infection, with minimal effect on the viral load and some histopathological findings. The difference between the present results and those of previous studies in younger animals^26^ flags the age of the target population as a relevant factor that impact on the success of prophylactic interventions. Both preclinical studies in geriatric models and clinical studies in the elderly population are needed to prove the efficacy of vaccination strategies initially designed for the general population, and to implement suitable adjustments that improve the efficacy of vaccination in elderly individuals.

Despite the initial SARS-CoV-2 vaccination campaigns were designed to use one or two vaccine doses (depending on the type of vaccine), the administration of multiple doses has been shown to improve vaccine immunogenicity and efficacy in elderly individuals^38^. Our results suggested that a longer interval between vaccine booster and SARS-CoV-2 exposure may increase neutralizing humoral responses against omicron variants which are difficult to neutralize by means of a shorter interval. Thus, the SARS-CoV-2 Omicron BA.1 heterologous neutralizing activity was improved in SARS-CoV-2 D614G challenged GSH that were immunized four months earlier compared with those that received the booster dose two weeks in advance. Interestingly, this improvement may be associated with neutralizing antibodies targeting other S epitopes than the RBD. Thus, to fine-tuning the gap between vaccine doses might contribute to increase the immunogenicity and efficacy of SARS-CoV-2 vaccine in the elderly population. Accordingly, increasing the gap between the first and the second vaccine dose (≥12 weeks) increased the efficacy of the ChAdOx1nCoV-19 vaccine^39^. Although it was not investigated in the present work, another parameter that would be worthy to be investigated during vaccine adaptation to elderly people is the amount of antigen. It has been documented that the mRNA-1273 induced higher titres of antibodies in elderly individuals than the BNT162b2 one, which might be explained by the higher amount of mRNA present in the former formulation (3-fold)^40^. In addition, the route of administration and the adjuvant also play a major role in vaccine efficacy. Recently, Jangra and co-workers^41^ have demonstrated that the lower immunogenicity observed with the use of recombinant protein plus AddaVax in aged mice can be bypassed by a intranasal immunization using a recombinant protein formulated in an oil in water nanoemulsion adjuvanted with a novel RNA-based RIG-I agonist (IVT DI). All these data, including the present study, highlight the importance to investigate in alternatives to current vaccine approaches to improve their efficacy in challenging groups such as elderly or immunocompromised individuals.

## Methods

### Virus isolates

SARS-CoV-2 isolates (GISAID ID EPI_ISL_510689, designed as Cat01, and GISAID ID EPI_ISL_47147, designed as Cat02) were used in this study. Both viruses were isolated from two human patients (oropharyngeal swab) in Spain in March and April 2020, respectively, and they are both characterised by the Spike mutation D614G^42^. Production of viral stocks, isolation, and titration was performed in Vero E6 cell (ATCC® CRL-1586™). Virus titres were determined using a standard TCID_50_ assay and expressed as TCID_50_/mL calculated according to the Reed-Muench method^43^.

### Animals

The study was performed using 26 advanced geriatric B6.Cg-Tg(K18-ACE2)2Prlmn/J (K18-hACE2) (22–23-months-old) mice from Jackson Laboratories (Bar Harbor, Maine, USA), and 43 geriatric (13–18-months-old) golden Syrian hamsters (GSH) from Envigo (Indianapolis, Indiana, USA)^28,29^. A balanced sex ratio was used for both species. Each individual animal was considered to be the experimental unit. Animals were allocated to the Centre for Comparative Medicine and Bioimage (CMCiB) for ageing. Immunisation, post-immunisation follow-up (both mice and GSH) and SARS-CoV-2 challenge of K18-hACE2 mice were also performed at CMCiB. GSH challenge was performed in the Biocontainment Unit of IRTA-CReSA. Animal experiments, conducted by certified staff, were approved by the Institutional Animal Welfare Committee of the *Institut de Recerca i Tecnologia Agroalimentàries* (IRTA) and of the Centre for Comparative Medicine and Bioimage (CMCiB, CSB20–015-M7) and by the Ethical Commission of Animal Experimentation of the Autonomous Government of Catalonia (codes: 10965 and 11094).

### In vivo immunogenicity and vaccine efficacy studies

To evaluate the immunogenicity and efficacy of the recombinant trimeric Spike 29 protein (S-29) against SARS-CoV-2 D614G isolates in geriatric animal models ten K18-hACE2 mice and 27 GSH were immunised subdermally with 15 µg of recombinant protein adjuvanted with AddaVax^TM^ (Invivogen) in the hock. Three weeks later, animals received a boosting dose in the hock of the other leg. Control animals (mice *n* = 9, GSH *n* = 12) were primed and boosted with PBS plus AddaVax ^TM^. Two weeks after boosting, 10 S-29-immunised and 9 control K18-hACE2 mice were challenged intranasally with 10^3^ TCID_50_ of SARS-CoV-2 (Cat01 isolate) per animal, receiving a total volume of 50 µL (25 µL/nostril). Of note, two S-29 immunised mice died at the time of challenge, probably due to the anaesthesia. Six unvaccinated mice remained as uninfected controls and were not challenged.

S-29 immunised GSH (*n* = 27) were distributed into two experimental groups: 1) SARS-CoV-2 challenged three weeks days after booster immunisation (*n* = 12, short-term group), and 2) SARS-CoV-2 challenged 4 months after booster immunisation (*n* = 15, long-term group). Four non-immunised/non-inoculated GSH were included as uninfected controls. Twelve GSH immunised only with adjuvant were challenged with SARS-CoV-2 (infected controls). GSH intranasal challenge was performed with 10^4^ TCID_50_ SARS-CoV-2 (Cat02 isolate) in a final volume of 100 µL (50 µL/nostril). Animals in the uninfected control groups received an equal volume of PBS intranasally.

Body weight and clinical signs were monitored daily after the viral challenge; weight loss higher than 20%, lack of response to stimuli, and lack of motility were set as humane endpoints (HEP). Four to five animals per experimental group were euthanised by intraperitoneal pentobarbital overdose under general anaesthesia (5% isoflurane) on days 2, 4, and 7 post-inoculation (dpi), except for the four animals of the uninfected control group, which were euthanised on 7 dpi. Random allocation ensuring a balanced sex ratio was used for both species. Only healthy animals were included in the study, and animals showing any sign of disease were excluded.

### Sample collection

After euthanasia, nasal turbinate (NT), lung, and brain (mice only) samples were collected and fixed by immersion in 10% buffered formalin for pathological analyses or placed into individual 1.5 mL tubes containing 500 µL of DMEM (GIBCO) supplemented with 1% penicillin-streptomycin (GIBCO), for molecular detection and viral titration purposes. For the latter purpose, tissue samples were homogenized at 30 Hz using a TissueLyser II (QIAGEN GmbH) 30 seconds. Blood samples were collected from each animal from either the facial vein (mice) or cava vein (GSH, under anaesthesia) before each immunization, and by cardiac puncture at euthanasia under deep anaesthesia, and left at room temperature for two hours for clotting. After that, serum was collected by centrifugation (10 minutes at 5000 *g* or 1000 *g* for mice and hamster samples, respectively). All samples were stored at −70 °C until use.

### RNA-extraction and quantitative RT–PCR

Genomic RNA (gRNA), indicating the presence of the virus in the tissue of interest, and subgenomic RNA (sgRNA), considered a proxy for viral replication^44^ were quantified by RT-qPCR. Since the animal studies were carried out in two different laboratories (mice: IrsiCaixa, hamsters: IRTA-CReSA), slightly different viral RNA isolation and RT-PCR methodologies were used. For mouse samples, viral RNA was extracted from target organs and swabs samples using the Viral RNA/Pathogen Nucleic Acid Isolation kit and a KingFisher instrument (ThermoFisher Scientific). PCR amplification of gRNA was based on the 2019-Novel Coronavirus Real-Time RT-PCR Diagnostic Panel guidelines and protocol developed by the American Center for Disease Control and Prevention using N2 primers and probes (2019-nCov CDC EUA Kit, Integrated DNA Technologies), and the GoTaq 1-Step RT-qPCR kit (Promega, Madison, WI, USA). Thermal cycling was performed at 50 °C for 15 min for reverse transcription, followed by 95 °C for 2 min, and then 45 cycles of 95 °C for 10 sec, 56 °C for 15 sec, and 72 °C for 30 sec in the Applied Biosystems 7500 or QuantStudio5 Real-Time PCR instruments (ThermoFisher Scientific). For absolute quantification, a standard curve was built using 1/5 serial dilutions of a SARS-CoV2 plasmid (2019-nCoV_N_Positive Control, 200 copies/μL, Integrated DNA Technologies) and run in parallel in all PCR determinations. The viral load of each sample was determined in triplicate and the mean viral load (in copies/mL) was extrapolated from the standard curve and corrected by the corresponding dilution factor.

For hamster samples, viral RNA was isolated using the IndiMag pathogen kit (Indical Bioscience) on a Biosprint 96 workstation (QIAGEN), according to the manufacturer’s instructions. RT-PCR used to detect viral gRNA is based on the protocol published by Corman et al.^45^, with minor modifications to adapt it to the AgPath-ID One-Step RT–PCR Kit (Life Technologies). RT-PCR targets a portion of the Envelope protein gene (position 26,141–26,253 of GenBank NC_004718). The sequences of the primers and probes used, and their final concentrations are as follows: Forward 5′-ACAGGTACGTTAATAGTTAATAGCGT-3′ [400 nM]; Reverse: 5′-ATATTGCAGCAGTACGCACACA-3′ [400 nM]; Probe: 5′-FAM-ACACTAGCCATCCTTACTGCGCTTCG-TAMRA-3′ [200 nM]. For absolute quantification of gRNA, a standard curve was built using 1/10 serial dilutions of a SARS-CoV2 plasmid (2019-nCoV_E Positive Control, 2×10^5^ copies/μL, Integrated DNA Technologies) and run in parallel in all PCR determinations. Results are expressed as log_10_ (copies/mL) of lysates or DMEM for tissue samples and OP swabs, respectively.

Both mouse and GSH viral sgRNA detection by RT–PCR was based on the protocol published by Wölfel et al.^46^ with minor modifications. The Reverse primer and probes are the same used for gRNA detection, while the sequence of the Forward primer is: 5′-CGATCTCTTGTAGATCTGTTCTC-3′ [400 nM]. Thermal cycling was performed at 55 °C for 10 minutes for reverse transcription, followed by 95 °C for 3 minutes and then 45 cycles of 95 °C for 15 seconds, and 56 °C for 30 seconds both for gRNA and sgRNA.

### Viral titration in Vero E6 cells

Supernatant from the homogenized lungs, NT, and brain (mice only) collected at different times after inoculation were evaluated for the presence of infectious virus by titration in Vero E6 cells as previously described^47,48^. Briefly, each sample was 10-fold diluted (10^−1^ to 10^−10^) in duplicates, transferred into a 96-well plate with a Vero E6 cells monolayer, and incubated at 37 °C and 5% CO2. Plates were monitored daily under an optical microscope and wells were evaluated for the presence or absence of cytopathic effect at 6 dpi. The amount of infectious virus was calculated by determining the TCID_50_ according to the Reed-Muench method^49^.

### Pathology and immunohistochemistry

Formalin-fixed upper (NT) and lower (lung) respiratory tract from both species, and brain samples from mice were processed for histopathology, and haematoxylin and eosin (H&E) stained slides were examined under an optical microscope. A semi-quantitative score based on the amount of inflammation and the severity of the observed lesions was assigned to each sample (0 = none, 1 = mild, 2 = moderate, 3 = severe). Score assignment by a European College of Veterinary Pathologists (ECVP) certified pathologist was performed in a blinded fashion^36,50^.

Immunohistochemistry was used to detect SARS-CoV-2 nucleoprotein using the rabbit monoclonal antibody (40143-R019, Sino Biological) at dilution 1:15,000, followed by an Envision+/HRP-conjugated anti-rabbit secondary antibody (K4003, Dako; ready-to-use solution, 90 µL/slide). A semi-quantitative score was assigned to each tissue sample, according to the amount and distribution of the nucleoprotein-specific staining (0= absence of viral antigen; 1= low amount, multifocal localisation; 2= moderate amount, multifocal localisation; 3= high amount, diffuse localization). Score assignment by an ECVP-certified pathologist was performed in a blinded fashion^36,50^.

### Immunological assays

Anti-Spike (S) and anti-receptor binding domain (RBD) IgG were quantified in serum samples using an in-house ELISA. SARS-CoV-2 Wuhan-Hu-1 S (cat# 40589-V08B1) or RBD recombinant proteins (cat# 40592-V08H) (Sino-Biological) were diluted in PBS at 1 µg/mL and added to one-half of a Nunc MaxiSorp ELISA plate and coated overnight at 4 °C, while the other half-plate was incubated with PBS only. After two hours of blocking with PBS/1% bovine serum albumin (BSA, Miltenyi biotech), heat-inactivated plasma samples were assessed in duplicates in wells containing either SARS-CoV-2 antigens or PBS/1%BSA. Mouse standards were prepared in blocking buffer, as a serial 1/3 dilution of the anti-6xHis antibody HIS.H8 (cat# MA1-21315-1MG, ThermoFisher Scientific), starting at 1 µg/mL. GSH standard was similarly prepared using a positive GSH serum starting at 1/100. Samples were diluted between 1/100 – 1/5000 in blocking buffer. After blocking, 50 µL of each standard or diluted sample were added to the plate and incubated overnight at 4 °C. The HRP conjugated (Fab)2 Goat anti-mouse IgG (Fc specific) (1/20,000) (cat# 115-036-071, Jackson Immunoresearch), or Goat anti-hamster IgG (H + L) (1/20,000) (cat# 107-035-142, Jackson Immunoresearch) were used as detection antibodies for mouse and GSH IgG determination, respectively. O-phenylenediamine dihydrochloride (OPD, Sigma Aldrich) was used as a substrate. The enzymatic reaction was stopped with 2 M H_2_SO_4_ (Sigma Aldrich). The signal was analysed using optical density at 492 nm with noise correction at 620 nm. The antigen-specific signal was calculated by subtracting the background obtained for each sample in antigen-free wells. Results are shown as arbitrary units (AU)/mL.

### Pseudovirus production and neutralisation assay

The neutralising activity of serum samples was assessed as previously described^51^. Briefly, pseudoviruses were produced by co-transfecting Expi293F cells (ThermoFisher Scientific) with the single-round HIV reporter pNL4-3.Luc.R-.E- (NIH AIDS Reagent Program^52^) and the SARS-CoV-2.SctΔ19 plasmid that codes for the Spike glycoprotein of the Wuhan (WH1) or the Omicron BA.1 variant, with a deletion of the last 19 C-term residues. As a control, the SARS-CoV-2 spike was replaced by VSV-G plasmid was used to generate VSV-G expressing HIV pseudoviruses. Transfections were performed using the ExpiFectamine293 Reagent kit (ThermoFisher Scientific). Supernatants were harvested after 72 hours, filtered at 0.45 μm, and frozen at -80 °C until use. Pseudoviruses were titrated on HEK293T cells overexpressing WT human ACE-2 (HEK293T/hACE2) (Integral Molecular, USA). After heat inactivation (56 °C for 60 minutes), 3-fold serial dilutions of serum samples (range 1/60–1/14,580) were incubated with 200 TCID_50_ of SARS-CoV-2 derived pseudoviruses for 1 hour at 37 °C. Then, 1×10^4^ HEK293T/hACE2 cells treated with DEAE-Dextran (Sigma-Aldrich) were added. After 48 hours, BriteLite Plus Luciferase reagent (PerkinElmer, USA) was added and plates were read in an EnSight Multimode Plate Reader. The serum samples’ neutralising activity was determined using a 4-parameters logistic equation in Prism 8.4.3 (GraphPad Software, USA) and showed as normalized ID_50_ (reciprocal dilution inhibiting 50% of the infection).

### Statistical analysis

Quantification of anti-SARS-CoV-2 IgG and neutralising antibody titers were compared between groups using the Kruskal-Wallis test, and longitudinally using the Friedman test. Post-hoc tests were performed using the corresponding Conover’s test. Weight variation and severe disease incidence in SARS-CoV-2-challenged animals were analyzed by mixed two-way ANOVA followed by Tukey’s multiple comparisons test, using the positive control of infection or the negative control group as a reference. Viral load (SARS-CoV-2 gRNA and sgRNA) and infectivity (log(TCID_50_) differences were analyzed with Peto&Peto left-censored samples test. Histopathology analysis was performed using an asymptotic generalized Pearson chi-squared test. In all cases, multiple comparisons were corrected by false discovery rate (FDR). P values lower than 0.05 were considered significant and are shown as follows: *p < 0.05, **p < 0.01, ***p < 0.001, ****p < 0.0001. Statistical analyses were completed utilizing R software (version 4.1) and GraphPad Prism v8.0.

### Reporting summary

Further information on research design is available in the Nature Research Reporting Summary linked to this article.

### Supplementary information

Reporting Summary

Supplementary material

## Data Availability

Row data generated in this study is available upon request.

## Code availability

No unreported custom computer code or algorithm has been used to generate or process the data presented in the manuscript.
