# Supplementary material for: Immunisation efficacy of a stabilised SARS-CoV-2 spike glycoprotein in two geriatric animal models
Source: NPJ Vaccines. 2024 Feb 27;9:48. doi: 10.1038/s41541-024-00840-0 (PMC10899648; doi:10.1038/s41541-024-00840-0)
Supplement: Supplementary file 2 — Supplementary material [file 41541_2024_840_MOESM2_ESM.pdf]

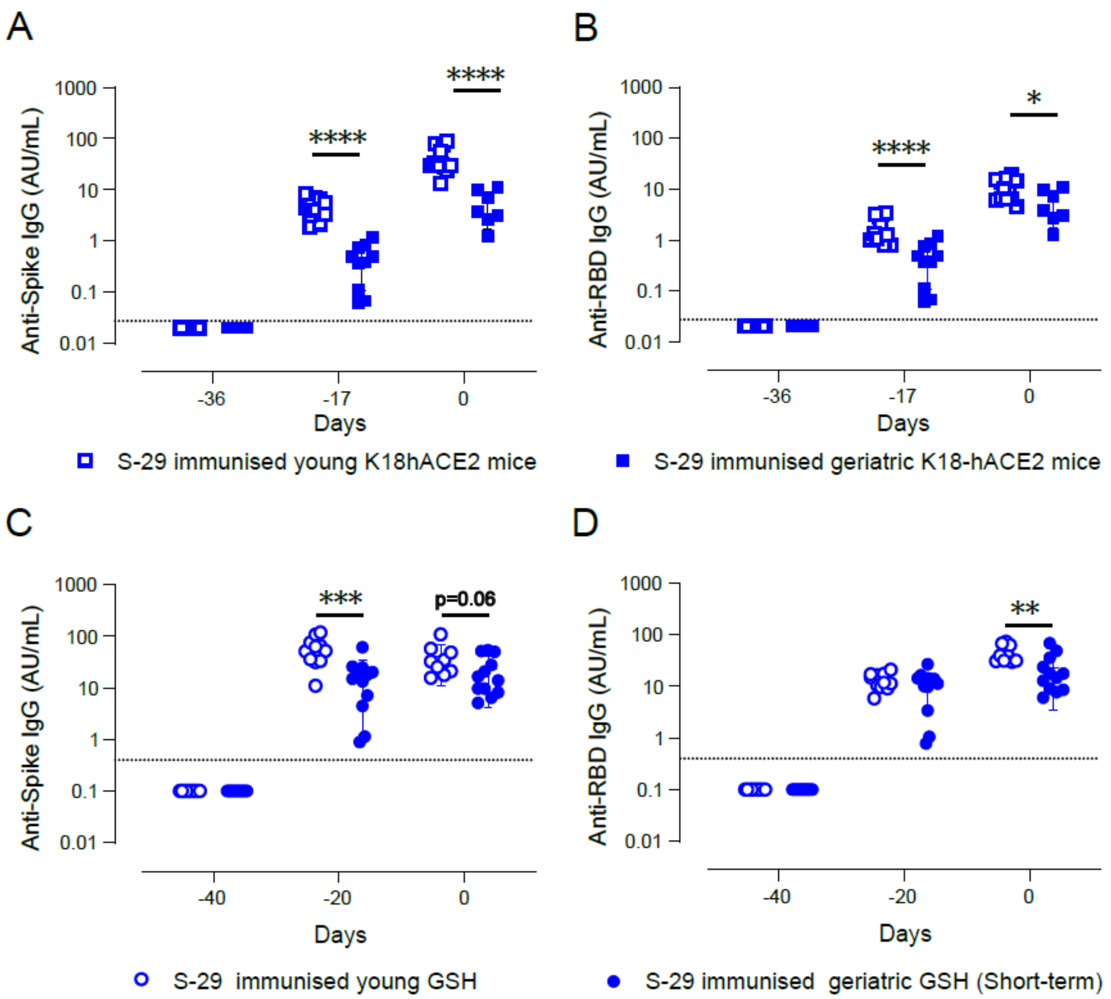

Supplementary Figure 1. Humoral immune kinetics (IgG against S and RBD) in both geriatric animal models. Kinetics of the humoral immune response progression in (A, B) K18-hACE2 geriatric mice and (C, D) geriatric GSH, in comparison with the humoral immune response elicited in young individuals. Single data points, mean and standard deviation are shown for each experimental group. Differences between groups were determined using the Kruskal-Wallis test, and longitudinally using the Friedman test. Post-hoc tests were performed using the corresponding Conover's test. P-values < 0.05 were considered significant; *: p-value < 0.05; ****: p-value < 0.0001.

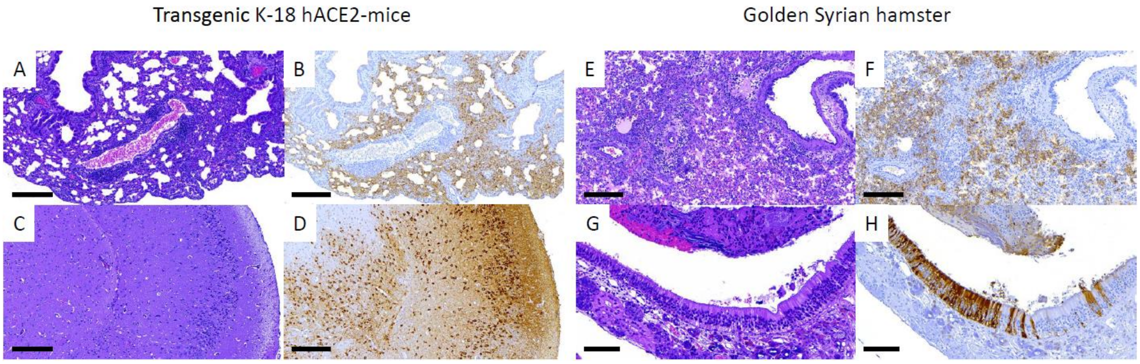

Supplementary figure 2. Pathological outcome examples of geriatric transgenic K18-hACE2 mice and golden Syrian Hamsters (GSH) inoculated with SARS-CoV-2. A) Mild broncho-interstitial pneumonia (score 1) in a Addavax treated control mouse. B) High amount of SARS-CoV-2 nucleoprotein (score 3) in the lung of the same mouse. C) Mild non-suppurative meningo- encephalitis (score 1) in a Addavax treated control mouse. D) High amount of SARS-CoV-2 nucleoprotein (score 3) in the brain of the same mouse. E) Moderate broncho-intersticial pneumonia (score 2) in a positive control GSH. F) High amount of SARS-CoV-2 nucleoprotein (score 3) in the lung of the same GSH. G) Mild non-suppurative rhinitis (score 1) in a S-29 vaccinated GSH. H) Moderate amount of SARS-CoV-2 nucleoprotein (score 2) in the nasal turbinates of the same GSH. Figures A, C, E and G: Hematoxylin & Eosin stain. Figure B, D, F and H: Immunohistochemistry for SARS-CoV-2 nucleoprotein with Haematoxylin counterstain. Bars of figures A to F: 200 μm; bars of figures G and H: 80 μm.
